# A Squeak

**Published:** 1872-04

**Authors:** 


### A SQUEAK

Is the most hateful noise in the world, in dozing in church, or in a sick chamber. Soaking the soles of the shoes in oil is not always efficacious; but a piece of oiled silk between each layer of leather in the sole is an infallible remedy.

